# COVID-19: anxiety among hospital staff and associated factors

**DOI:** 10.1080/07853890.2020.1862905

**Published:** 2020-12-22

**Authors:** Elina Mattila, Jaana Peltokoski, Marko H. Neva, Marja Kaunonen, Mika Helminen, Anna-Kaisa Parkkila

**Affiliations:** a Administration Centre, Tampere University Hospital, Tampere, Finland; b Central Finland Health Care District, Jyväskylä, Finland; c Department of Orthopaedic and Trauma Surgery, Tampere University Hospital, Tampere, Finland; d Faculty of Social Sciences, Tampere University, Tampere, Finland; e Tays Research Services, Tampere University Hospital, Tampere, Finland; f Faculty of Social Sciences, Health Sciences, Tampere University, Tampere, Finland

**Keywords:** COVID-19, anxiety, hospital staff

## Abstract

**Background:**

During the COVID-19 pandemic, hospital staff have experienced a variety of mental health challenges. European research on anxiety and stress among hospital workers during the pandemic is limited. This study aimed to describe the anxiety levels of Finnish hospital workers during the COVID-19 pandemic.

**Methods:**

The multidimensional, cross-sectional survey was distributed to all hospital staff working at two Finnish specialized medical care centres in the spring of 2020 (*n* = 1,995). The Generalized Anxiety Disorder 7-item (GAD-7) scale was used to measure the workers’ anxiety.

**Results:**

The total mean GAD-7 score was 4.88, indicating normal anxiety levels. However, 30% (*n* =  1,079) of the respondents had mild, 10% (*n* = 194) moderate and 5% (*n* = 88) severe anxiety. Key risk factors were young age, working in a university hospital, problems in cooperation between co-workers, difficulty concentrating at work, a health-threatening physical and psychological workload, and a fear of being infected at work.

**Conclusion:**

Hospital staff experienced a variety of work-related stress and anxiety issues that should be visible to hospital administrators and policymakers alike. The anxiety is independent of whether the worker is directly involved in caring for or in any way coming into contact with COVID-19 patients.

**Key message**
- Fifty-five percent of hospital staff have normal anxiety levels. The remaining workers may need targeted support interventions, and a smaller proportion (15%) are in danger of developing longer-term problems affecting their well-being. The anxiety experienced by hospital workers during the COVID-19 pandemic is more severe than that of the population on average. If the pandemic continues, the well-being of hospital staff may be widely threatened. Despite the different geographical locations and COVID-19 situations, hospital workers in Finland and China had similar anxiety levels.
- The anxiety is independent of whether staff are working in the front line of managing the COVID-19 pandemic or of the number of covid-19 patients admitted to the hospital. The hospital workers felt anxiety because they were facing a new situation which causes changes in their work and daily routine. Health care employers should engage in long-term follow-up as regards the personnel’s recovery from the burden caused by the pandemic and from work in general. It is necessary to make easily attainable, flexibly delivered and cost-effective treatment interventions for anxiety available to hospital staff.

## 1. Introduction

Since the first confirmed case of COVID-19 was announced at the end of 2019, the coronavirus disease has become a global health emergency [1,2]. This unprecedented pandemic has caused several global challenges for both the health care system and health care staff [3,4]. Worldwide, a great deal of attention has focussed on vaccine development and on how the health care system is coping with the outbreak of COVID-19 [5,6]. However, the discussion on how health care staff have coped with the distress caused by the pandemic has received less attention [7,8].

Health care professionals in different clinical units work under extreme pressure, and it appears that working in the front line is a significant risk factor for psychological problems [9–11]. However, regardless of the place of work, an overwhelming workload and feelings of being insufficiently supported, as well as ethical concerns and conflict, can exacerbate professionals’ distress and anxiety [8,12,13]. Recent studies [14–16] showed that professionals treating patients infected with the new coronavirus experienced high rates of several symptoms, such as tiredness, insomnia and headaches. Moreover, Buselli et al. [9] showed that burnout and secondary traumatization were associated with health care workers’ depression or anxiety scores. In addition, Chew et al. [16] found that health care workers with physical symptoms were more likely to have depression, stress and anxiety. Psychological distress has also been reported to associate with physical symptoms, such as respiratory symptoms. This association is most probably bi-directional, which may result in the exacerbation of insignificant symptoms [16].

The psychological distress may be increased because professionals are worried about being infected and they may be afraid of transmitting the infection to family members, colleagues or patients [15,17]. Furthermore, a shortage or even lack of different types of personal protective equipment (PPE) may contribute to the anxiety and distress [8,17–19]. Especially for nurses, the pandemic may change their regular job duties, increase working overtime and cause them to be treated differently because of working in a hospital [10]. A recent study [20] observed that, in stressful situations caused by the COVID-19 pandemic, nurses cared for and helped each other and, to some extent, felt collective empowerment. However, nurses also activated psychological defence mechanisms, such as isolation and depression [10,20]. Moreover, Louie et al. [17] conducted a study on health professionals, particularly on spine surgeons. The results among the surgeons showed moderately high anxiety levels [17]. However, there are other personnel working in health care institutions who are not all medically trained, and, interestingly, they have even been shown to be at a higher risk of psychological distress than medically trained staff [21,22].

Perceived social support and precautions are measures that have been identified to reduce psychological distress [10]. As the pandemic continues, mental health support, encouragement and a sense of purpose are needed to support health care workers [13,14], in addition to continuing education related to coping and resilience training [9,18]. To ensure effective infection control measures, Tan et al. [21] emphasize the importance of educational interventions also among non-medical health care workers.

Currently (November 2020), the number and incidence of coronavirus cases have risen in Finland, especially among younger age groups and young adults [23]. However, people over 70 years of age remain in the high-risk group because of their age, and protecting them is a priority [23]. In relation to the Finnish population(5,543,233), the prevalence of cases (November 2020) is 309 and the incidence of new cases in relation to the population is 48,5 cases/100,000 inhabitants [23]. To date (November 2020), a total of 17,119 positive cases have been recorded in Finland, and a total of 361 deaths related to the disease have been reported. The current (November 2020) virus testing capacity is roughly 20,000 tests per day [23]. The COVID-19 epidemic is monitored with functional, epidemiological and medical indicators in terms of Finland’s hybrid strategy; the aims are to protect the capacity of the health care system, to prevent the spreading of the virus and to protect people, especially those who are most at risk [23].

Research on anxiety among hospital staff during the COVID-19 pandemic is limited and scant. The published studies on the COVID-19 pandemic have mainly dealt with the situation in Asia. To the best of our knowledge, this is the first European study concerning anxiety among health care workers during the COVID-19 pandemic. Therefore, to address this gap and to react to the increasing levels of stress before the next waves of the pandemic, it is urgent to understand the anxiety experienced by hospital staff during the COVID-19 pandemic in the Nordic countries.

## 2. Aim

The aim of the study is to describe the anxiety levels of Finnish hospital workers during the COVID-19 pandemic. A further objective is to determine the associations of background variables (demographic data, changes in the work, availability of personal protective equipment [PPE], interaction between workers, psychological distress, fears) with hospital workers’ anxiety levels.

## 3. Methods

### 3.1. Sample and data collection

The multidimensional, cross-sectional survey was distributed to all health care staff (*N* = 10,425) at Tampere University Hospital and Central Finland Central Hospital in Finland, covering a total catchment area of roughly 775,000 inhabitants. Finnish university hospitals are tertiary referral centres in which all medical specialties are represented, and they are tasked with providing more specialized and demanding treatment than central hospitals. If a central hospital cannot provide the appropriate treatment, a patient may be transferred from a central to a university hospital.

The data was collected between 24 April and 12 May 2020 while emergency conditions were in force in Finland (e.g. schools and borders were closed). The hospital staff responded to the survey anonymously *via* the Webropol system. The total sample size is 1995, yielding a response rate of 19%.

Most of the participants were women (*n* = 1,731, 87%), had a degree from a university of applied sciences (*n* = 991, 50%) and were regular employees (*n* = 1,558, 79%). The largest age group was those aged 31–40 years (*n* = 522, 26%). The majority of the respondents were working at the university hospital (80%, *n* = 1,605) and belonged to the nursing staff (*n* = 1,302, 66%). The smaller group consisted of physicians (*n* = 121, 6%) and other hospital staff (administration, services and psychologists, logopaedists, occupational therapists, dieticians, chemists; 28%, *n* = 565). The background information is shown in Table 1.

**Table 1.** The GAD-7 anxiety scale’s associations with the hospital workers’ background variables.

Background variable	GAD-7 total score		
	Mean	SD	*p* Value
Gender			
Female (n = 1,731, 87%)	5.07	4.78	<.001
Male (n = 255, 13%)	3.46	4.43	
Occupational group			
Physician (n = 121, 6%)	3.04	3.57	<.001
Nursing staff (n = 1,302, 66%)	5.46	4.90	
Other (n =565, 28%)	3.90	4.39	
Age			
18–30 (n = 389, 20%)	6.03	5.16	<.001
31–40 (n = 522, 26%)	5.53	4.85	
41–50 (n = 503, 25%)	4.48	4.54	
51–55 (n = 277, 11%)	4.12	4.12	
56– (n = 351, 18%)	3.65	4.33	
Type of hospital			
University hospital (n = 1,605, 80%)	5.08	4.83	<.001
Central hospital (n = 390, 20%)	4.05	4.15	
Educational level			
University (n = 329, 17%)	3.45	3.81	<.001
University of applied sciences (n = 991, 50%)	5.62	5.0	
Other (n = 664, 33%)	4.46	4.61	
Employment			
Regular (n = 1,558, 79%)	4.74	4.65	.022
Temporary (n = 413, 21%)	5.38	5.10	
Work experience (years)			
0–3 (n = 605, 30%)	5.17	4.95	<.001
4–10 (n = 510, 26%)	5.45	4.97	
11–20 (n = 510, 26%)	4.59	4.46	
21– (n = 364, 18%)	3.77	4.27	
Manager duty			
Yes (n = 200, 10%)	3.06	3.52	<.001
No (n =1,778, 90%)	5.08	4.83	

### 3.2. Study instruments

The Generalized Anxiety Disorder 7-item scale (GAD-7) was used to measure health care workers’ anxiety during the COVID-19 outbreak. The GAD-7 is a self-report scale developed to assess the defining symptoms of anxiety [24]. The items are rated on a 4-point Likert-type scale (from 0 = *not at all* to 3 = *nearly every day*), and the scores range from 0 to 21. The GAD-7 has been used in earlier COVID-19 studies [5,8], which facilitates the comparison of the results. The reliability and validity of the GAD-7 instrument has been demonstrated in earlier studies [25,26]. In the current study, the Cronbach’s alpha coefficient of the GAD-7 was 0.92.

In addition to the six background factors, the questionnaire also comprised the six groups of items. These were: 1) workers’ demographic data (gender, age, type of hospital, education level, occupational group, employment, work experience and managerial duty; Table 1); 2) changes in work (remote work during the COVID-19 outbreak [yes/no], transfer to another work unit [yes/no], Table 2); 3) current and future availability of personal protective equipment (PPE) (yes, uncertain, no; Table 2); 4) interaction between co-workers (cooperation between peers has been smooth [yes, uncertain, no], team spirit has been good [yes, uncertain, no], cooperation between different professions has been smooth [yes, uncertain, no], Table 3); 5) psychological distress (increase in work-related stress [yes, uncertain, no], increased difficulty concentrating at work [yes, uncertain, no], increased work-related thoughts outside working hours [yes, uncertain, no], and increased workload that threatens physical and psychological health [yes, uncertain, no], Table 3); and 6) fears (afraid of being transferred to a new work unit [yes, uncertain, no], afraid of being infected at work [yes, uncertain, no], afraid of transmitting the virus to family members [yes, uncertain, no], Table 3).

**Table 2.** GAD-7 anxiety scale’s associations with the changes in work and availability of personal protective equipment (PPE) during the COVID-19 pandemic.

Background variable	GAD-7 total score		
	Mean	SD	*p* Value
Changes in work			
Remote work			
Yes (n = 294)	3.39	3.81	<.001
No (n = 1,696)	5.14	4.86	
Transferred to a new work unit			
Yes (n = 345)	6.82	5.61	<.001
No (n = 1,641)	4.45	4.40	
Availability of personal protective equipment (PPE)			
Enough available at the moment			
Yes (n = 1,377, 70%)	4.72	4.62	<.001
Uncertain (n = 360, 18%)	4.36	4.52	
No (n = 235, 12%	6.79	5.52	
Enough available enough if pandemic continues			
Yes (n = 911, 46%)	3.90	4.19	<.001
Uncertain (n = 672, 34%)	4.99	4.67	
No (n = 390, 20%)	7.11	5.41	

**Table 3.** GAD-7 anxiety scale’s associations with interaction between workers, psychological distress and fears during the COVID-19 pandemic.

Background variable	GAD-7 total score		
	Mean	SD	*p* Value
Interaction between workers			
Cooperation between co-workers was smooth			
Yes (n = 1,741, 89%)	4.53	4.47	<.001
Uncertain (n = 79, 4%)	6.77	5.75	
No (n = 130, 7%)	8.51	6.06	
Team spirit at work was good			
Yes (n =1,698, 85%)	4.44	4.46	<.001
Uncertain (n = 110, 6%)	6.03	5.07	
No (n = 183, 9%)	8.25	5.68	
Cooperation between professions			
was smooth			
Yes (n =1,624, 82%)	4.50	4.42	<.001
Uncertain (n = 197, 10%)	5.13	5.07	
No (n = 156, 8%)	8.57	5.93	
Psychological distress			
Work-related stress increased			
Yes (n = 1,188, 60%)	6.97	4.79	<.001
Uncertain (n = 84, 4%)	2.66	2.08	
No (n = 720, 36%)	1.69	2.46	
Difficulty concentrating at			
work increased			
Yes (n = 652, 33%)	8.66	5.11	<.001
Uncertain (n = 180, 9%)	5.22	3.68	
No (n = 1,157, 58%)	2.70	3.05	
Thinking about work-related matters			
outside working hours increased			
Yes (n = 1,173, 60%)	6.83	4.86	<.001
Uncertain (n = 65, 3%)	3.81	3.15	
No (n = 752, 37%)	1.95	2.83	
Workload threatened physical			
and psychological health			
Yes (n = 537, 27%)	9.60	5.15	<.001
Uncertain (n = 270, 14%)	5.54	3.44	
No (n = 1,180, 59%)	2.60	2.85	
Fears			
Afraid of being transferred to another work unit			
Yes (n = 764, 38%)	7.25	5.15	<.001
Uncertain (n = 137, 7%)	4.06	4.49	
No (n = 1,091, 55%)	3.30	3.69	
Afraid of contracting the coronavirus at work			
Yes (n = 719, 36%)	7.29	5.07	<.001
Uncertain (n = 173, 9%)	5.13	4.45	
No (n = 1,097, 55%)	3.27	3.84	
Afraid of infecting a family member			
Yes (n = 1,085, 55%)	6.51	4.94	<.001
Uncertain (n = 108, 5%)	4.25	4.78	
No (n = 796, 40%)	2.76	3.50	

Because a ready-made questionnaire was not available for these six items, the questions were developed for this study. The questions were pretested with hospital workers (*n* = 10) prior to the data collection, and based on the pretesting, the wording of some questions was clarified prior to the data collection.

### 3.3. Statistical analysis

The data were analysed statistically with SPSS 25 software, with a p value of <.05 indicating statistical significance [27]. Frequencies, percentage distributions and means were used as descriptive analysis methods. In addition, standard deviation and range (min., max.) were used to describe the data.

According to the earlier studies, the GAD-7 scale was divided into four anxiety categories: normal (0–4.99), mild (5–9.99), moderate (10–14.99) and severe (15–21) [5,8,11,22]. For the logistic regression model, the anxiety levels were divided into two categories: no/mild anxiety (GAD score 0–9.99) and moderate/severe anxiety (GAD score ≥ 10) [5].

The associations between anxiety scores and background factors were analysed using non-parametric Mann–Whitney *U* tests and Kruskal–Wallis tests. For the purposes of the analysis, the participants were categorized into five age groups: 18–30, 31–40, 41–50, 51–55 and ≥ 56 years. Work experience was also categorized into four groups: 0–3, 4–10, 11–20 and ≥ 21 years of experience (Table 1).

Multivariable logistic regression models with the enter method were used to examine the factors independently associated with anxiety (GAD score ≥ 10). These independent factors are shown in Tables 1–3. The associations between background risk factors and anxiety are presented as odds rations (OR) and 95% confidence intervals (95% CI).

### 3.4. Ethical considerations

According to Finnish guidelines, a review by an institutional ethics committee was not required for this study. Permission to conduct the research was obtained from the directors of both hospitals. Participation in the study was voluntary. Hospital staff were informed of the study in writing. All data were treated confidentially, and results were reported in such a way that it is not possible to identify the respondents.

## Results

### 4.1. The hospital workers’ level of anxiety

The total mean GAD-7 score was 4.88 (SD 4.75, range 0–21), indicating a normal anxiety level in the whole sample. For 55% (*n* = 1,079) of the workers, the anxiety level was normal, while 30% (*n* = 587) had mild anxiety, 10% (*n* = 194) had moderate anxiety, and 5% (*n* = 88) had severe anxiety.

### 4.2. Association of background factors to the anxiety of hospital workers

#### 4.2.1. Hospital staff demographic data

Female respondents had more anxiety than male participants (mean 5.07 vs 3.46, *p* < .001), and nursing staff had more anxiety than physicians or other staff (mean 5.46 vs 3.04 and 3.90, respectively, *p* < .001). The youngest workers (aged 18–30 years) reported the highest (mean 6.03) and those aged over 56 years the lowest anxiety levels (mean 3.65, *p* < .001 for age group differences). The university hospital staff had higher anxiety levels than those working at the central hospital (mean 5.08 vs 4.05, *p* < .001). The workers with a degree from a university of applied sciences had more anxiety than those with a university or other degree (mean 5.62 vs 3.45 and 4.46, respectively, *p* < .001). The anxiety levels were higher amongst temporary staff than regular staff (mean 5.38 vs 4.74, *p* < .001), and subordinates reported higher levels than managers (mean 5.08 vs 3.06, *p* < .001). Those who had been working in the hospital for 4–10 years had the highest anxiety levels when compared to others (mean 5.45 vs 5.17 and 4.59 and 3.77, *p* < .001) (Table 1).

#### 4.2.2. Changes in the work

Administrative staff were encouraged to work from home as much as possible to help to keep the physical distance to avoid spreading the disease. Amongst workers, 15% (*n* = 294) were working remotely full-time or part-time, whilst 85% were working full-time in the hospital (Table 2). Out of those who responded to the questionnaire, 17% (*n* = 345) were transferred to work in another unit because of the pandemic. The workers who had worked remotely during the COVID-19 outbreak had less anxiety than those who had not (mean 3.39 vs 5.14, *p* < .001). Furthermore, the workers who had moved to a new unit had more anxiety than those who stayed in the same unit (mean 6.82 vs 4.45, *p* < .001) (Table 2).

#### 4.2.3. Availability of personal protective equipment (PPE)

The majority (71%, *n* = 1,377) of the workers thought that there had been enough personal protective equipment (PPE), such as surgical masks, available in their hospital during the COVID-19 outbreak. However, 46% (*n* = 911) of the participants believed that there would not be enough PPE if the COVID-19 pandemic continues (Table 2). The workers who had enough personal protective equipment (PPE) available in their units had less anxiety than those who experienced a shortage of PPE (mean 4.72 vs 6.79, *p* < .001). Also, those who believed that there would be enough PPE in the future experienced less anxiety (mean 3.90 vs 7.11 *p* < .001) (Table 2).

#### 4.2.4. Interaction between workers

The majority of the respondents (90%, *n* = 1,780) felt that the collaboration between co-workers had been smooth during the pandemic. Furthermore, 85% (*n* = 1,696) of the participants estimated that the team spirit had been good during the outbreak. Also, 82% (*n* = 1,624) of the staff felt that the cooperation between different professions had been smooth (Table 3). Good cooperation between co-workers (mean 4.53 vs 8.51, *p* < .001) and good team spirit in the work community (mean 4.44 vs 8.25, *p* < .001) reduced the experiences of anxiety. Workers who felt that the cooperation between different professions had been smooth had less anxiety (mean 4.50 vs 8.57 *p* < .001) (Table 3).

#### 4.2.5. Psychological distress

More than half (60%, *n* = 1,188) of the participating hospital staff felt that their work-related stress had increased during the COVID-19 outbreak. Furthermore, a third of the respondents (33%, *n* = 652) had experienced more difficulties concentrating at work during the pandemic. Over half of the participating staff (60%, *n* = 1,173) expressed increased work-related thoughts during their free time. Also, 28% (*n* = 537) of the respondents felt that the workload had increased during the COVID-19 outbreak to the degree that it threatened their physical and psychological health (Table 3). The workers who felt that work-related stress had increased during the COVID-19 outbreak had more anxiety than those whose stress levels had not increased (mean 6.97 vs 1.69 *p* < .001). Staff members who had experienced increased difficulty concentrating during the COVID-19 pandemic also had more anxiety (mean 8.66 vs 2.70 *p* < .001). The workers who were thinking about work-related issues outside working hours more than earlier had higher anxiety levels than others (mean 8.83 vs 1.95 *p* < .001). Furthermore, in individuals whose work-related physical and psychological burden was increased to a level that threatened the individual’s health, the anxiety levels were the highest (mean 9.60 vs 2.60, *p* < .001) (Table 3).

#### 4.2.6. Fears

More than one third of the staff (38%, *n* = 763) were afraid that they would be transferred to a new work unit during the COVID-19 outbreak. Furthermore, 37% (*n* = 719) feared that they would contract the coronavirus while at work and 55% (*n* = 1,085) that they would infect a family member (Table 3). A fear of being transferred to another unit during the pandemic was associated with more anxiety than not being afraid of transfer (mean 7.25 vs 3.30, *p* < .001). Furthermore, the workers who were afraid of being infected with COVID-19 at work had more anxiety than those who were not afraid of the infection (mean 7.29 vs 3.27, *p* < .001). Anxiety was also associated with a fear of transmitting the virus to family members (mean 6.51 vs 2.76, *p* < .001) (Table 3).

### 4.3. Risk factors of hospital worker anxiety

According to the logistic regression analysis, the independent risk factors that predicted the hospital workers’ anxiety during the COVID-19 outbreak were age (*p* = .012), type of hospital (*p =* .012), cooperation between co-workers (*p* = .002), difficulty concentrating at work (*p* < .001), physical and psychological workload that threatens a person’s health (*p* < .001) and a fear of contracting the COVID-19 infection while at work (*p =* .002) (Table 4). The hospital workers’ probability of experiencing anxiety decreased with age (≥ 56 years OR = 0.31, 95% CI 0.13–0.74, *p* = .009, vs 18–30 years). The staff at the university hospital were more likely to experience anxiety than those working at the central hospital (OR = 2.15, 95% CI 1.18–3.93, *p =* .012). Furthermore, the hospital workers who had experienced problems in cooperation between co-workers were more likely to have anxiety than those who experienced smooth cooperation (OR = 3.11, 95% CI 1.64–5.90, *p* = .001). Work-related difficulty concentrating increased the likelihood of having anxiety (OR = 3.34, 95% CI 2.07–5.39, *p* < .001). The hospital workers who experienced that the work-related physical and psychological burden threatened their health were more likely to have anxiety than those who had not been over-burdened (OR = 7.59, 95% CI 4.48–12.85, *p* < .001). Furthermore, workers who were afraid of being infected with COVID-19 in their work were more likely to have anxiety than those who were not afraid of being infected (OR = 2.23, 95% CI 1.34–3.71, *p* = .002) (Table 4). The other background variables shown in Tables 1–3 did not independently predict the hospital workers’ anxiety.

**Table 4.** Logistic regression model to predict variables associated with anxiety (GAD-7 ≥ 10)*.

	Anxiety		
	OR	[95% CI]	*p* Value
Age			
18–30	1.00		0.012
31–40	0.94	[0.55–1.60]	.83
41–50	0.44	[0.23–0.86]	.016
51–55	0.38	[0.17–0.86]	.021
56–	0.31	[0.13–0.74]	.009
Type of hospital			
Central hospital	1.00		
University hospital	2.15	[1.18–3.93]	0.012
Cooperation between co-workers was smooth			
Yes	1.00		0.002
Uncertain	1.30	[0.57–2.96]	.52
No	3.11	[1.64–5.90]	.001
Difficulty concentrating at work increased			
No	1.00		<0.001
Uncertain	1.51	[0.73–3.10]	.52
Yes	3.34	[2.07–5.39]	.001
The physical and psychological workload was health-threatening			
No	1.00		<0.001
Uncertain	1.46	[0.75–2.83]	.25
Yes	7.59	[4.48–12.85]	<.001
Afraid of contracting the coronavirus at work			
No	1.00		0.002
Uncertain	1.02	[0.49–2.13]	.95
Yes	2.23	[1.34–3.71]	.002

Total number of cases included in the model was 1749. The model Naqelkerke R Square was 51.6%.

*Only statistically significant variables are show in Table 4; in total, the model includes the following variables (Tables 1–3): gender, occupational group, age, type of hospital, education level, employment, work experience, manager duty, remote work, transferred to a new work unit, current and future availability of personal protective equipment, smoothness of cooperation between peers, team spirit, smoothness of the cooperation between different professionals, work-related stress, difficulty concentrating, work-related thoughts outside working hours, increased workload that threatens physical and psychological health, afraid of transfer to a new work unit, afraid to being infected, afraid of transmitting the virus to family members.

## 4. Discussion

To the best of our knowledge, this is the first study to examine anxiety and associated factors among Nordic hospital staff during the COVID-19 pandemic. Previous European research is also limited [28].The earlier studies in which anxiety has been examined have focussed on Asian hospital staff [5,8,10,11,14]. Finnish hospital workers’ anxiety levels (obtained using the GAD-7 instrument) seem to be similar to those of Chinese hospital staff and the wider Asian population [8,10,11,14,29,30]. In Finland, the COVID-19 pandemic has not been extremely severe, and the majority of the workers did not have direct contact with COVID-19 patients. Therefore, it is surprising that the hospital workers’ anxiety was quite similar to the experiences on the other side of the word in a quite different COVID-19 disease situation. It can be concluded that the workers’ anxiety is not directly associated with the number of COVID-19 patients and personally caring for them, but is rather related to a new and unexpected situation which causes changes in hospital routines and protocols. The fear of an unknown threat can also cause acute anxiety. This is in parallel with the results of the study by Xu et al. [31].

According to the present study, 55% of Finnish hospital workers have normal anxiety levels, but 30% have mild anxiety, and 10% suffer from moderate anxiety levels. The results show that 5% of Finnish hospital workers have suffered from severe anxiety (GAD ≥ 15) during the COVID-19 pandemic. The incidence of general anxiety disorder (GAD) in a Finnish population is 1.3% and in international materials 1.8–5.1% [32–34]. The COVID-19 pandemic seems to have increased anxiety symptoms among hospital workers who do not necessarily have a pre-existing psychiatric condition [35]. According to the results of this study, 60% of the participating Finnish hospital workers felt that work-related stress had increased during the COVID-19 outbreak. During the earlier SARS outbreak and the current COVID-19 pandemic, work-related stress and post-traumatic stress disorder (PTSD) among health care staff has varied between 13,8% and 80% [36–41], which leads us to conclude that the stress levels among Finnish hospital staff are high, taking into consideration the low numbers of COVID-19 patients.

As in earlier studies, female respondents had more anxiety than male participants in the current study [8,28,29,42,43]. The nursing staff had more anxiety than physicians and other non-medical hospital staff. The same result has been shown in earlier studies [8,11,18,29,43–45]. However, Tan et al. [21] and Chew et al. [22] reported that non-medically trained health care workers had a higher prevalence of anxiety than medical staff. Further research is needed to establish the reason for the difference in anxiety between different occupational groups. Naushad et al. [45] stated that nurses may develop more emotional bonding with the patients than physicians. The majority of the nursing staff are women, and they care for the patients in the front line, facing a death and difficult ethical dilemmas [10,36,45,46]. The physicians are better able to accept the risks related to the work than the nursing staff [36,47]. It is also possible that the physicians suppress their psychological symptoms more than the nurses and can underestimate their symptoms. For example, Elbay et al. [28] showed that 51.6% of Turkish physicians suffered from anxiety during the COVID-19 outbreak and that the symptoms were serious in 22% of the cases.

According to the results of the present study, a work-related fear of being infected was the independent factor to predict anxiety. Other studies have also highlighted that the fear of contracting the disease generates anxiety and stress [46]. As we stated earlier, the number of COVID-19 patients in Finland has been low, and this fear is thus not based on a realistically high risk of being infected while at work, but rather on the unfamiliar nature of the situation. The current results also show that the majority (70%) of the respondents felt that they had had enough personal protection equipment (PPE) available in their hospital so far. It is important to know that uncertainty about the availability of PPE is associated with increased anxiety. Workforce safety needs to be a high priority, and hospitals need to provide a safe working environment and sufficient protective supplies [46].

Our study shows that older workers expressed less anxiety than younger ones. Only a few studies have presented parallel results [28,48]. Higher age usually correlates with more work experience and confidence in being able to manage the work in a new situation. Working in a university hospital as opposed to a central hospital increased the workers’ anxiety levels. Lai et al. [8] reported similar results from Chinese hospitals. One explanation could be that a university hospital requires highly specialized expertise and skill to manage complex treatments and procedures, which can entail that the staff is more sensitive to changes in their work than central hospital staff.

The results of this study showed that a large number of hospital staff might need some targeted interventions and professional help in managing their anxiety. It is known that anxiety and psychological distress can have long-term effects, and mental health care support is important during the current pandemic to attenuate the escalation of psychological complications [14,35,49]. A lack of social support and communication, weak coping strategies and a lack of training can be factors leading to the development of mental health problems [45]. The effects of the COVID-19 pandemic on health care professionals span across the globe. When prolonged, anxiety and stress may lead to a deterioration of working ability and thereby to health-related absences and premature retirement. It is possible that the most significant costs of the COVID-19 pandemic will not be incurred by the treatment of COVID-19 patients, but will rather consist of the long-term effects of addressing the mental health of health care personnel and the population at large. Health care employers should engage in long-term follow-up as regards the personnel’s recovery from the burden caused by the pandemic and from work in general. The present study showed that 60% of the staff reported that they were thinking about work in their free time more than before the COVID-19 pandemic. It seems that the COVID-19 outbreak has obscured the boundary between work and free time among hospital staff. This probably complicates the recovery from work-related stress. The current results also showed that 33% of the hospital staff felt that work-related difficulty concentrating had increased during the COVID-19 pandemic. We observed that such difficulties are independently associated with anxiety. This is worrying because it can lead to mistakes and serious accidents at work.

The COVID-19 pandemic has required flexibility and resilience from hospital workers. They had to prepare for a severe epidemic and, therefore, establish cohort wards, extra intensive care units and separate pathways for infected patients. This required great efforts to educate staff in new ways of working, as well as transfers to other units. The results of this study show that changes in the working environment, or the mere threat of changes, can increase anxiety. Workers who were afraid of being transferred had significantly more anxiety than those who were not afraid of the changes. It is probable that the COVID-19 pandemic has shaken the hospital workers’ sense of security.

Regardless of the severity of the anxiety, the threshold for health care professionals to seek help for their anxiety may be high. The staff tend to be dutiful to the detriment of their own psychological health [46]. However, it is necessary to make easily attainable, flexibly delivered and cost-effective treatment interventions for anxiety available to hospital staff. Internet-based support interventions, as well as those implemented *via* the Moodle learning environment or a smart phone, are more cost-effective than face-to-face interventions [35,49,50]. The interventions should, however, be jointly approved by mental health care experts and the employers, in addition to being easily implementable [35,51]. Workers suffering from mild anxiety may benefit from low-threshold peer support. Stress management and relaxation techniques might yield benefit by improving coping ability and resilience [45].

Peer support is associated with the psychological well-being of the staff and should not be underestimated [13,28,52]. The logistic regression analysis included in this study showed that the extent and nature of cooperation between co-workers during the COVID-19 outbreak was an independent predictor of anxiety. Good cooperation decreased the staff’s anxiety. Those suffering from more severe anxiety (GAD ≥ 10) need targeted short- and long-term interventions. Studies have shown cognitive behaviour therapy (CBT) and mindfulness-based therapy (MBT) to be effective in managing stress and anxiety, in learning relaxation techniques, and in concentrating on the present [16,22,35,49,50,53]. CBT can also mitigate maladaptive coping, such as avoidance and self-blame [35]. As few as seven online sessions of CBT intervention significantly decrease immediate perceived stress and improve the prevention of severe psychiatric disorders, such as PTSD and depression [49].

This study has some limitations. Responding to the survey was voluntary, which has led to selection bias. The sample size was adequate, but the generalizability of the results is limited by the high non-response rate. All participants answered the questionnaire anonymously *via* the Webropol system, and it was not possible to send reminders, as we were not able to trace non-responders. Physicians were underrepresented, which renders the results regarding physicians tentative and ungeneralizable. The study design was cross-sectional, showing the hospital workers’ anxiety at the time of participating in the study. We do not have data on the workers’ the pre-existing anxiety levels before the COVID-19 pandemic nor on any other possible reasons (e.g. past psychiatric or other medical history) besides the COVID-19 outbreak which were causing anxiety and difficulties in coping with challenging situations. Future follow-up studies are needed, as well as further control of variables that may be connected to the anxiety.

## 5. Conclusion and implications for practice

It is obvious that the COVID-19 outbreak caused problems as regards the anxiety levels of hospital workers. The anxiety may be a global reaction amid the COVID-19 outbreak, at least among the female and nursing staff. However, an important observation is that anxiety is not dependent on whether the staff member is directly involved in caring for COVID-19 patients or on the number of COVID-19 patients admitted to the hospital. The hospital workers felt anxiety because they were facing a new situation and an unknown threat that caused changes in their work and daily routine. The anxiety experienced by hospital workers during the COVID-19 pandemic is more severe than that of the population on average. A large group (15%) are in danger of developing longer-term problems that affect their well-being, and they need a targeted intervention. Those at risk comprise young workers and staff working at a university hospital, in addition to those for whom the pandemic has caused difficulties concentrating, conflict and burdening at work, as well as a fear of contracting the disease at work. Employers should offer personnel easily attainable and flexibly delivered treatment interventions for anxiety, which are jointly approved by mental health care experts. Interventions relying on cognitive behaviour therapy (CBT) and mindfulness-based therapy (MBT) are recommended in the treatment of anxiety among hospital staff. Employers should continue to follow up on the staff’s psychological recovery from the COVID-19 pandemic even after the pandemic is over. It is also important to assess how the anxiety experienced by hospital workers in similar crises can be recognized and the necessary support interventions arranged as quickly and smoothly as possible.
